# Characterization of Toxin Genes and Antimicrobial Susceptibility of *Staphylococcus aureus* Isolates in Fishery Products in Iran

**DOI:** 10.1038/srep34216

**Published:** 2016-10-03

**Authors:** Noushin Arfatahery, Abolfazl Davoodabadi, Taranehpeimaneh Abedimohtasab

**Affiliations:** 1 Dev of Microbiology, Dept of Pathobiology, School of Public Health and Institute of Public Health Research, Tehran University of Medical Sciences, Tehran, Iran; 2 Microbiology Department, Medical School, Babol University of Medical Science, Babol, Iran

## Abstract

*Staphylococcus aureus* is one of the most common causes of seafood-borne diseases worldwide, which are attributable to the contamination of food by preformed enterotoxins. In this study, a total of 206 (34.3%) *Staphylococcus aureus* strains were obtained from 600 fish and shrimp samples and were tested for their antimicrobial susceptibility. We assessed the prevalence of the genes responsible for the staphylococcal enterotoxins (SEA, SEB) and toxic shock syndrome toxin 1 (TSST-1) genes. The results indicated that 34% of aqua food samples were contaminated with *S. aureus*, and 23.8% of these isolates were *mec-A*-positive. Sixty-four percent of the strains isolated from contaminated seafood was enterotoxigenic *S. aureus*, and 28.2% of SEs were MRSA-positive. The most prevalent genotype was characterized by the presence of the *sea* gene (45.2%), followed by the *seb* gene (18.5%), and the *tst* gene encoding TSST-1 was found in eight strains (3.9%). Of the 206 *S. aureus* isolates, 189 strains (84.9%) were resistant to at least one antibiotic. Given the frequent outbreaks of enterotoxigenic MRSA, it is necessary to make revisions to mandatory programmes to facilitate improved hygiene practices during fishing, aquaculture, processing, and sales to prevent the contamination of fishery products in Iran.

A major global cause of food poisoning, is caused by heat-stable staphylococcal enterotoxins (SEs) produced by enterotoxigenic strains of *Staphylococcus aureus*^1^. TSST-1 was the first toxin shown to be involved in toxic shock syndrome^2^. Enterotoxins and toxic shock syndrome toxin 1 (TSST-1) are members of the pyrogenic toxin super antigen (PTSAg) family^3^,^4^. *Staphylococcus aureus* is considered one of the most common pathogens responsible for outbreaks of food poisoning^1^,^4^. The widespread use of antibiotics has led to the emergence of multidrug resistant strains, which makes it more difficult to eradicate the diseases they cause and increases their incidence^5^. The increasing prevalence of antimicrobial-resistant *S. aureus* plays an important role in food safety and is a threat to healthcare systems^6^. Specifically, the emerging antimicrobial resistance of *S. aureus* has become a major public health concern^7^,^8^,^9^. The number of resistant MRSA strains is increasing^6^,^7^,^8^, and there have been reports of MRSA in aquatic animals^10^. These studies have raised additional food safety concerns regarding *S. aureus* beyond its role as an agent that causes food poisoning^9^. Previous food safety research on *S. aureus* has focused on characterizing SE production; however, relatively little is known about the antimicrobial susceptibility profiles of enterotoxigenic *S. aureus* strains or the prevalence of SE and other virulence factors among MRSA isolates^9^. Given the frequent outbreaks of enterotoxigenic MRSA, new hygiene policies and management practices should be adopted to increase food safety and avoid extra treatment costs^9^. A number of researchers in other countries have investigated the potential transmission of this dangerous strain to food products by human carriers, the environment, activities such as transport and packaging, the contaminated hands of workers, or infected respiratory secretions^5^,^6^,^7^,^8^,^9^,^10^. Illness caused by enterotoxigenic MRSA is usually self-limiting but occasionally can be sufficiently severe to cause hospitalization^11^. The real incidence of staphylococcal food poisoning (SFP) is underestimated, primarily due to misdiagnosis, its sporadic nature, or minor outbreaks that go unreported^12^. For example, 29 cases of poisoning were underestimated in the European Union in 2008, primarily due to misdiagnosis; however, outbreaks were indeed reported, affecting 414 persons, of which 26 patients required hospitalization^12^. In the United States, SFP is estimated to account for 185,060 illnesses and 1,753 cases requiring hospitalization annually^13^. Some studies have reported MRSA in aquatic animals and identified antimicrobial-resistant *S. aureus* as the causative agent in cases of fish handlers’ disease^14^,^15^,^16^,^17^,^18^. Accordingly, a variety of techniresh marine shrimp sd be employed to survey for the presence of MRSA and related toxins in foodstuff 9. Currently, molecular biology techniques are considered important tools in microbiological studies^9^,^10^,^11^. Molecular typing of *S. aureus* strains plays a crucial role in epidemiological studies examining disease origins and can be used to monitor major incidents of contamination^6^,^8^. The aim of the present study was to determine the antimicrobial susceptibilities of *S. aureus* isolates derived from various fish and shrimp samples in Iran from 2013 to 2014. It also aimed to characterize methicillin-resistant, toxigenic *S. aureus* isolates by evaluating their ability to produce the *mec-A*, *sea, seb,* and *tst* genes with polymerase chain reaction analysis (PCR). The final aim of this study was to investigate the contamination of fishery products before their purchase and consumption.

## Materials and Methods

### Full ethics statement

The authors are reporting experiments carried out on fish and shrimp fishery products; experiments were not performed with live vertebrates or humans.

The experiments were conducted in accordance with the guidelines of the National Committee for Clinical Laboratory Standards (CLSI 2011). 19-Clinical and Laboratory Standards Institute Performance Standards for Antimicrobial Susceptibility Testing; Twenty-First Informational Supplement. CLSI document M100-S20. Clinical and Laboratory Standards Institute, Wayne, PA 2011 (date of access: January 2011).

All experimental protocols were approved by the Institute of Standards & Industrial Research of Iran (ISIRI). NO: 6806-1 Microbiology of food and animal feeding stuffs – Enumeration of coagulase – Positive *Staphylococci* (*Staphylococcus aureus* and species) technique using Baird–Parker agar medium 2006 (19/09/2006).

This work was supported by Tehran University of Medical Sciences and the Management Program (240.31) of the Public Health Faculty.

The experiments were conducted as recommended by the Institute of Standards and Industrial Research of Iran^19^,^20^.

### Sampling

In this cross-sectional study, a total of 600 samples (fresh and frozen, farm and marine), including 150 marine shrimps, 150 farmed shrimps, 150 farmed fish, and 150 marine fish with a healthy appearance were selected from September 2013 to September 2014.

The shrimp samples were caught from the south seas of Iran (Persian Gulf, Oman Sea, Indian Ocean) and, together with aquaculture-farmed shrimps, were brought to the Tehran fishery.

Fish were caught from the North Sea of Iran (Caspian Sea) and the south seas of Iran (Persian Gulf, Oman Sea, Indian Ocean) and, together with aquaculture-farmed fish, were brought to the Tehran fishery. All seafood samples were prepared in the Central Fish Market at the Tehran fishery. Each week, 12 samples were obtained for this study. Samples were transferred under standard conditions (in sterile container carriers at a temperature of 4 °C for fresh samples and −18 °C for frozen samples) from the fishery to the laboratory at Tehran University of Medical Sciences, generally within approximately 2 hours. After receiving the samples, diagnostic tests were immediately performed.

### Isolation and identification of *S. aureus*

One gram of each sample was mixed with 9 CCs of Gulity (*S. aureus* selective enrichment medium; Merck, Darmstadt, Germany) containing 0.1% potassium tellurite in suspension. Tubes were incubated at 37 °C for 24–48 hours. Tubes that contained deposits or were black were cultured on Baird-Parker agar (Merck, Darmstadt,Germany) containing an egg-yolk tellurite emulsion (Merck, Darmstadt, Germany), which was used for isolation^5^,^21^,^22^. Isolates were identified employing the following criteria: production of coagulase, DNase, catalase, mannitol fermentation, and a haemolytic zone on 5% sheep’s blood agar (Merck); additionally, a VP test and Gram staining were performed. *S. aureus* ATCC 25923 and *S. aureus* ATCC 29213 were used in tests as a negative control and a positive control, respectively.

### Antimicrobial susceptibility testing

The resistance of staphylococcal isolates to methicillin was tested via disk diffusion assay using Müeller-Hinton agar as recommended by the guidelines of the National Committee for Clinical Laboratory Standards (CLSI 2010)^23^. Methicillin-resistant isolates were recognized via the Kirby-Bauer disk diffusion method^8^ using cefoxitin-impregnated (MAST, UK) disks on Müeller-Hinton agar (Merck, Darmstadt, Germany) and commercially available disks (MAST, UK) supplemented with 2% NaCl for MRSA isolates. The following antibiotic disks were tested: penicillin, oxacillin, ampicillin, gentamicin, ciprofloxacin, erythromycin, clindamycin, chloramphenicol, vancomycin, tetracycline, and rifampin. Suspensions equivalent to 0.5 McFarlands were cultured on MHA and incubated at 35 °C for 18–24 h. Inhibition zones were measured and interpreted as recommended by the guidelines of the Clinical and Laboratory Standards Institute (CLSI 2010)^23^.

### DNA Extraction

DNA was isolated using a Viogene kit (Taiwan) as recommended by the manufacturer; however, 25 λ/ml lysostaphin was added to the bacterial suspensions. The extracted DNA was resolved via the electrophoresis of 5 ml of product on a 1% (w/v) agarose gel stained with ethidium bromide in TAE buffer at 100 V for 25 min and visualized with a gel documentation system (Bio-Rad).

### Primer Design

To subtype MRSA and the toxins produced by each strain, PCR was carried out employing several primer sets. The oligonucleotide primers used in this study were described by Johnson *et al*.^24^,^25^ and the protocol for MRSA subtyping was reported by Vannuffel *et al*.^26^. Table 1 lists the primer sets used to detect two SE genes (*sea, seb*), the TSST-1 gene (*tst*), and MRSA (*mec-A*).

### PCR amplification

#### PCR for Staphylococcal Enterotoxins (SEA, SEB) and TSST-1

PCR reaction mixtures contained 20 ng of template DNA, 1 U *Taq* DNA polymerase, 250 mM of each dNTP, 10 mM Tris-HCl (pH 9.0), 40 mM KCl, and 1.5 mM MgCl_2_ (Bioneer, Korea)^25^. Amplifications were carried out in a thermal cycler (Primus 96 advanced, USA) with the following thermal program: initial denaturation for 5 min at 95 °C; annealing for 30 cycles of 1 min at 95 °C, 1 min at 53 °C, and 2 min at 72 °C; final extension for 5 min at 72 °C.

#### PCR for MRSA

The isolated strains were also subjected to a PCR assay to detect the *mec-A* gene using a primer pair (Table 1) that was previously reported by Vannuffel *et al*.^26^. PCR amplification was carried out in a thermal cycler (Primus 96 advanced, USA) with initial denaturation at 94 °C for 5 min, followed by 35 cycles of denaturation at 94 °C for 30 s, primer annealing at 60 °C for 30 s, and extension at 72 °C for 30 s. A final extension was performed at 72 °C for 5 min^8^.

All PCR products were resolved by the electrophoresis of 5 ml of product on a 1% (w/v) agarose gel stained with ethidium bromide in TAE buffer at 100 V for 25 min and visualized with a gel documentation system (Bio-Rad).

### Statistical analysis

A chi-square test was used to compare the prevalence of each gene profile among *S. aureus* isolates between categories (SPSS 19). Differences between the prevalence rates were considered significant when p < 0.05.

## Results

A total of 206 *S. aureus* isolates were grouped into eight categories based on seafood sample origin, i.e., aqua products (fresh marine shrimp, frozen marine shrimp, fresh farm shrimp, frozen farm shrimp; n = ~300) and fish (fresh marine fish, frozen marine fish, fresh farm fish, frozen farm fish; n = ~300). As shown in Table 2, a total of 84 (28%) out of 300 shrimp samples were found to be contaminated with *Staphylococcu*s *aureus*. Analysis revealed that 18 (24%) fresh marine shrimp samples, 30 (40%) frozen marine shrimp samples, 15 (20%) fresh farm shrimp samples, and 21 (28%) frozen farm shrimp samples were contaminated with *S. aureus*. Chi-square test, p < 0.05 (Table 2). A total of 122 (40.7%) out of 300 fish samples were found to be contaminated with *Staphylococcu*s *aureus*. The analysis revealed that 37 (49.3%) fresh marine fish samples, 53 (70.7%) frozen marine fish samples, 12 (16%) fresh farm fish samples, and 20 (26.7%) frozen farm fish samples (n = ~300) were contaminated with *S. aureus.* Chi-square test, p < 0.05 (Table 3).

A total of 206 *S. aureus* isolates demonstrated different antimicrobial resistance profiles, and 17 (14.1%) out of the 206 strains were susceptible to all tested compounds. Overall, the isolates were observed to primarily be resistant to β-lactams such as penicillin 163 (79.1%) and ampicillin 163 (79.1%); however, no samples were resistant to chloramphenicol, ciprofloxacin, rifampin, or vancomycin. The remaining strains were resistant to three or more classes (here considered multidrug resistant) (104, 56.3%) or two classes (54, 28.6%) of antimicrobials. The frequencies of resistance to individual agents were 49 (23.8%) for oxacillin, 7 (3.4%) for erythromycin, 5 (2.4%) for clindamycin, 58 (28.1%) for tetracycline, and 10 (4.8%) for gentamicin. Chi-square test, p < 0.05 (Table 4)

Over 64% of *S. aureus* isolates (n = 133) carried enterotoxin genes. As shown in Table 5, the majority of the strains included in this study carried the *sea* gene (95, 45.2%) and the *seb* gene (38, 18.5%); a small number of strains carried *tst* (8, 3.9%). Moreover, only nineteen (9.2%) isolates carried both the *sea* and *seb* enterotoxin genes. In two (0.97%) isolates, the *sea*, *seb*, *tst* and *mec-A* genes were detected.

A methicillin susceptibility test indicated that forty-nine (23.8%) *S. aureus* isolates were resistant to this antibiotic. All isolates carrying the *mec-A* gene demonstrated positive MRSA phenotypes, specifically 3 (16.7%) fresh marine shrimp samples, 5 (16.7%) frozen marine shrimp samples, 7 (46.6%) fresh farm shrimp samples, and 8 (38%) frozen farm shrimp samples. Furthermore, *S. aureus* isolates were MRSA-positive in 6 (16.2%) fresh marine fish samples, 7 (13.2%) frozen marine fish samples, 5 (41.7%) fresh farm fish samples, and 8 (40%) frozen farm fish samples, all carrying the *me-cA* gene (chi-square test, p < 0.05).

In total, one hundred and thirty-three (64%) of the strains isolated from contaminated fishery products were enterotoxigenic *S. aureus*, and 5.2% of SEs were MRSA-positive (p < 0.05).

## Discussion

It is difficult to detect pathogens; thus, good hygiene is very important. The quality and safety of fish and shrimp can be directly influenced by the lack of hygienic habits of fish handlers and contact with contaminated work surfaces, including benches, tables and unwashed knives^14^,^27^. Various factors impinge upon seafood safety, ranging from contamination originating from the environment where it is caught to contamination caused by the consumer prior to eating^16^,^28^. The most frequently contaminated types of seafood in our study were frozen marine fish and frozen marine shrimps. This contamination might have originated from contaminated freezing systems or the improper transfer of aqua products from their origin to their destination. Delays in product transport, worker non-compliance with food hygiene standards, improper storage conditions such as incompatible temperature, and the accumulation of fish or shrimp can result in greater amounts of contamination than fresh products. Furthermore, farmed seafood products were less contaminated compared with other types of products that were improperly frozen and packed. Microbial growth is affected by environmental factors such as pH, temperature, and water activity. In this study, resistance to penicillin, amoxicillin, oxacillin, and tetracycline between fresh and frozen products was not equivalent (Table 4). In addition, a statistically significant relationship was observed between the *sea*^+^ and *mec-A*^+^ genotypes and fresh and frozen seafood as follows: fresh products were more frequently positive for the *Staphylococcus aureus* gene *sea*, while the *mec-A*^+^ genotype was observed more frequently in frozen products.

In addition, *S. aureus* was detected in fish at levels as high as those observed in staphylococcal food poisoning cases derived from raw fish reported in many countries^14^,^15^,^28^.

In Japan, the foods that are most frequently involved in staphylococcal food poisoning are sushi (raw fish) and lunch-box meals that contain multi-ingredient foods^29^,^30^. In Italy, *S. aureus* is primarily detected in meat products^3^. The major foods involved in outbreaks in Korea from 1996 to 2000 were meat, raw and undercooked sea food, multi-ingredient foods, and lunch-box meals^31^. When RTE (ready-to-eat) food contamination was monitored in Korea, it was determined that 19.8% of raw fish was contaminated with *S. aureus*^8^,^28^.

In a similar study carried out with aqua products in India, the rate of *S. aureus* contamination was 17%, which is much lower than our findings^27^. Other researchers have reported lower rates of *S. aureus* than our results^8^,^16^,^28^,^29^. A study examining marine products in Brazil reported a high rate of contamination with *S. aureus*^10^,^14^. One of the primary reasons for variations in these results may be the impact of environmental pollution as well as differences in the processing of fishery products due to maintenance.

Antibiotic resistance can be spread via residual antibiotics in food products, through the transfer of resistant foodborne pathogens, or through the ingestion of resistant strains among original food microflora and the transfer of resistance to pathogenic microorganisms^32^. In our study, the rate of resistant strains was high (91.8%). Resistance to members of the penicillin family was 79% for penicillin and 78.6% for ampicillin. Among *S. aureus* food isolates in Portugal, resistance to erythromycin and tetracycline was 5% and 7%, respectively^33^, whereas in a study in Botswana (Loeto *et al*.)^34^, resistance rates to erythromycin and tetracycline were 18.6% and 50.5%, respectively, among isolates collected from food handlers^34^. A study in Brazil reported high rates of *S. aureus* contamination in marine products. In that study, isolates were resistant to ampicillin, and 44% were multidrug resistant^14^.

In our study, frozen farmed fish were found to be more frequently contaminated than other types of fish. Moreover, fresh farmed shrimp were found to be more frequently contaminated than other shrimps. This might be attributable to the inappropriate use of antibiotics in aquaculture.

Zhang *et al*.^35^ showed that multidrug resistance is common among MRSA isolates, which is not surprising due to their ability to transfer staphylococcal cassette chromosome *mec-A* elements and additional resistance determinants on plasmids^35^. Among *S. aureus* seafood isolates in Spain^21^, samples were analysed for the presence of *Staphylococcus aureus*^36^. *S. aureus* was detected in a significant proportion of products (~25%). All isolates were resistant to penicillin, chloramphenicol and ciprofloxacin, and most were resistant to tetracycline (82.4%), but none was methicillin-resistant.

In our study, the *mec-A* gene was detected in 23.8% of *S. aureus* isolates (49 out of 206). The high prevalence of resistance genes should be considered a potential health risk for humans and seafood^37^. Consequently, mandatory precautions should be taken by governments and individuals to prevent the further spread of MRSA. Hygiene promotion and avoiding the unsupervised use of antibiotics are elementary steps in this regard^1^,^37^. On the other hand, the investigation of strains for enterotoxin production is important in pathogenesis studies^38^. In our study, 5.2% of MRSA isolates were positive owing to the presence of SEs.

With the exception of ninety-five (45.2%) isolates, all SE-carrying isolates identified in this study carried the *sea* gene and thus could produce SEA, but thirty-eight (18.5%) isolates were found to be *seb*-positive. Classical staphylococcal enterotoxins (SEA–SEE) have been reported to cause 95% of staphylococcal food poisoning. Among them, SEA is the most common in staphylococcus-related food poisoning cases^39^,^40^,^41^, likely due to its very high resistance to proteolytic enzymes^1^. In a study performed by Sanchez *et al*. in Spain^31^, strains isolated from 23.5% of samples were enterotoxigenic. A lower proportion of *SEs and tsst-1*-positive strains were previously found among isolates collected from fishery products in other geographical regions^3^,^10^,^25^. The *sea*-only genotype and the *sea* genotype in combination with other genes are the most prevalent genotypes in Japan (80% to 90%) and in the United States (50%)^40^,^41^. In 2011, an outbreak of gastroenteritis at the Athletic Club in Barcelona, Spain was studied, and the results showed that 91 people had been infected via the ingestion of contaminated fish. The infectious *Staphylococcus aureus* strains produced enterotoxin types A and D^28^.

In 2007, Kérouanton studied a total of 178 coagulase-positive staphylococcal isolates recovered from 31 staphylococcal food-poisoning outbreaks (SFPO) 1981–2002 that were screened through biotyping^42^. Eleven strains were resistant to at least two classes of antibiotics, and among them, two were resistant to methicillin. Twenty-nine strains carried one or several of the eight SE genes tested; the *sea* gene was the most common gene (n = 23) and was often linked to *sed* (n = 12) or *seh* (n = 5).

The results of this study show that enterotoxigenic *S. aureus* that is resistant to antibiotics can cause dangerous epidemics and outbreaks. In brief, detection of the SE genes using molecular techniques may help to understand the pathogenic potential of this microorganism^37^.

## Conclusions

The results of our study highlight the high prevalence of enterotoxigenic MRSA in fishery products, and the risk of its transmission through the food chain cannot be disregarded^6^,^12^, particularly in uncooked meat and raw shrimp and fish. Moreover, this study provides further support for the hypothesis that MRSA can undergo cross-contamination between humans and the environment. Our results showed that the increasingly non-standard use of antibiotics in fish and shrimp aquaculture can lead to *S. aureus* resistance. As a result, the transmission of resistant bacteria from fish and shrimp to humans is a consequence of antibiotic overuse.

Despite advances in food research, contamination remains a problem and a major cause of mortality and morbidity worldwide. Our results showed that seafood monitoring requires urgent attention, although more research is needed to verify these results.

## Additional Information

**How to cite this article**: Arfatahery, N. *et al*. Characterization of Toxin Genes and Antimicrobial Susceptibility of *Staphylococcus aureus* Isolates in Fishery Products in Iran. *Sci. Rep.* **6**, 34216; doi: 10.1038/srep34216 (2016).

## Figures and Tables

**Table 1:** PCR primers and fragment lengths of the studied genes.

Target gene	Primer	Oligonucleotide sequence (5′ −3′)	Size (bp)
*tst*	F	CATCTACAAACGATAATATAAAGG	481
	R	CATTGTTATTTTCCAA TAACCACCC	
*sea*	F	CCTTTGGAAACGGTTAAAACG	127
	R	TCTGAACCTTCCCATCAAAAAC	
*seb*	F	TCGCATCAAACTGACAAACG	477
	R	GCAGGTACTCTATAAGTGCCTGC	
*mec-A*	F	GAA ATG ACT GAA CGT CCG AT	399
	R	CTG GAA CTT GTT GAG CAG AG	

**Table 2:** Distribution of *S. aureus* in the shrimp samples analyzed.

Presence of Staphylococcus aureus			
Studied samples	Number of non-contaminated samples	Number of contaminated samples	Number of samples
Fresh marine shrimp	57 (76.0%)	18 (24.0%)	75 (100%)
Frozen marine shrimp	45 (60.0%)	30 (40.0%)	75 (100%)
Fresh farmed shrimp	60 (80.0%)	15 (20.0%)	75 (100%)
Frozen farmed shrimp	54 (72.0%)	21 (28.0%)	75 (100%)
Total	216 (72.0%)	84 (28.0%)	300 (100%)

**Table 3:** Distribution of *S. aureus* in the fish samples analyzed.

Presence of *Staphylococcus aureus*			
Studied samples	Number of non-contaminated samples	Number of contaminated samples	Number of samples
Fresh marine fish	38 (50.7%)	37 (49.3%)	75 (100%)
Frozen marine fish	22 (29.3%)	53 (70.7%)	75 (100%)
Fresh farmed fish	63 (84.0%)	12 (16%)	75 (100%)
Frozen farmed fish	55 (73.3%)	20 (26.7%)	75 (100%)
Total	178 (59.3%)	122 (40.7%)	300 (100%)

**Table 4:** Antimicrobial resistance of foodborne *S. aureus* isolates.

Samples (no. of isolates)	No. (%)of isolates resistant to:										
	P	OX	AM	GM	CIP	E	C	VA	CC	TE	RA
Fresh marine shrimp (18)	16 (88.9^*^)	3 (16.7)	16 (89.9)	0	0	0	0	0	0	0	0
Frozen marine shrimp (30)	19 (63.3)	5 (16.7)	19 (63.3)	0	0	0	0	0	0	0	0
Fresh farmed shrimp (15)	10 (66.7)	7 (46.6)	11 (73.3)	1 (6.7)	0	0	0	0	0	4 (26.6)	0
frozen farmed shrimp (21)	14 (66.6)	8 (38)	14 (66.6)	1 (4.8)	0	0	0	0	0	4 (19)	0
Fresh marine fish (37)	34 (91.9)	6 (16.2)	33 (89.1)	0	0	0	0	0	0	17 (46)	0
Frozen marine fish (53)	41 (77.3)	7 (13.2)	41 (77.3)	0	0	2 (3.7)	0	0	2 (3.7)	9 (17)	0
Fresh farmed fish (12)	12 (100)	5 (41.7)	12 (100)	3 (25)	0	2 (16.7)	0	0	2 (16.7)	10 (83)	0
Frozen farmed fish (20)	17 (85)	8 (40.0)	17 (85)	5 (25)	0	3 (15)	0	0	1 (5)	14 (70)	0
Total Avg (206)	163 (79.1)	49 (23.8)	163 (79.1)	10 (4.8)	0	7 (3.4)	0	0	5 (2.4)	58 (28.1)	0

*P: penicillin, OX: oxacillin, AM: ampicillin, GM: gentamicin, CIP: ciprofloxacin, E: erythromycin, CC: clindamycin, VA: vancomycin, C: chloramphenicol, TE: tetracycline, RA: rifampin.

*The numbers in parentheses indicate the percentages.

**Table 5:** Presence of the studied *Staphylococcus aureus* genes.

Studied genes of *Staphylococcus aureus*	Genes: positive		Genes: negative		Total	
	No.	Percent	No.	Percent	No.	Percent
*sea*	95	45.2%	111	54.8%	206	100%
*seb*	38	18.5%	168	81.5%	206	100%
*tst-1*	8	3.9%	198	96.1%	206	100%
*mec-A*	49	23.8%	157	76.2%	206	100%
*sea* + *seb*	19	9.2%	187	90.8%	206	100%
*sea* + *seb* + *mec-A*	7	3.4%	199	96.6%	206	100%
*sea* + *seb* + *mec-A* + *tst-1*	2	0.97%	204	99%	206	100%
